# Clinical outcomes of Atezolizumab Therapy for Previously-Treated Advanced-Stage Non-Small Cell Lung Cancer: A Real-World Study in Taiwan

**DOI:** 10.7150/jca.74617

**Published:** 2022-07-18

**Authors:** Shang-Gin Wu, Chi-Lu Chiang, Chin-Chou Wang, Jen-Yu Hung, Te-Chun Hsia, Chih-Hsi Kuo, Jin-Yuan Shih

**Affiliations:** 1 Department of Internal Medicine, National Taiwan University Hospital, National Taiwan University, Taipei, Taiwan.; 2 Department of Internal Medicine, National Taiwan University Cancer Center, National Taiwan University, Taipei, Taiwan.; 3 Department of Chest Medicine, Taipei Veterans General Hospital, Taipei, Taiwan.; 4 School of Medicine, National Yang Ming Chiao Tung University, Taipei, Taiwan.; 5 Institute of Clinical Medicine, National Yang Ming Chiao Tung University, Taipei, Taiwan.; 6 Division of Pulmonary and Critical Care Medicine, Department of Medicine, Chang Gung Memorial Hospital-Kaohsiung Medical Center, Chang Gung University College of Medicine, Kaohsiung, Taiwan.; 7 Department of Internal Medicine, Kaohsiung Municipal Ta-Tung Hospital, Kaohsiung Medical University, Kaohsiung, Taiwan.; 8 Division of Pulmonary and Critical Care Medicine, Department of Internal Medicine, Kaohsiung Medical University Hospital, Kaohsiung Medical University, Kaohsiung, Taiwan.; 9 Faculty of Medicine, College of Medicine, Kaohsiung Medical University, Kaohsiung, Taiwan.; 10 Division of Pulmonary and Critical Care Medicine, Department of Internal Medicine, China Medical University and China Medical University Hospital, Taichung, Taiwan.; 11 Division of Lung Cancer and Interventional Bronchoscopy, Department of Thoracic Medicine, College of Medicine, Chang Gung Memorial Hospital, Chang Gung University, Taipei, Taiwan.

**Keywords:** Atezolizumab, Epidermal growth factor receptor mutation, Non-small-cell lung cancer, Tyrosine kinase inhibitor, Osimertinib, Immune checkpoint inhibitor

## Abstract

Immune checkpoint inhibitors (ICIs) are the standard treatment for non-small-cell lung cancer (NSCLC). We assessed the clinical prognostic factors in NSCLC patients receiving atezolizumab as a second- or later-line (2L+) treatment. Data were retrospectively collected for NSCLC patients treated with atezolizumab from July 2017 to June 2019 at six medical centers in Taiwan. Clinical characteristics, treatment course and responses of patients were recorded. A total of 128 NSCLC patients received 2L+ atezolizumab, and the outcomes included a response rate of 10.2%, median progression-free survival (mPFS) of 3.5 months, and median overall survival (mOS) of 10.7 months. Eleven patients who had received osimertinib treatment before atezolizumab had a shorter mPFS (2.3 *versus* 3.5 months; *p* = 0.002) and mOS (4.8 *versus* 11.2 months; *p* < 0.001) than those without prior osimertinib treatment. Even for the subgroup of patients with *EGFR*-mutant non-squamous NSCLC, prior osimertinib was still associated with shorter PFS (2.3 *versus* 4.1 months; *p* = 0.006) and OS (4.8 *versus* 11.7 months; *p* < 0.001). Multivariate analysis revealed that prior osimertinib treatment correlated with not only shorter PFS (hazard ratio [HR]: 2.94; 95% confidence interval [CI], 1.34-6.47; *p* = 0.007) but also shorter OS (HR, 3.55; 95% CI, 1.57-8.03; *p* = 0.002). Patients with prior ICIs treatment (HR, 3.18; *p* = 0.002) or poor performance status (HR, 2.70; *p* = 0.001) had shorter OS. In conclusion, osimertinib treatment before atezolizumab therapy was associated with a shorter PFS and a poor prognosis in NSCLC patients in real-world settings. Further studies with larger sample sizes are needed to validate these observations.

## Introduction

Lung cancer is the leading cause of cancer-related deaths 1. Traditionally, platinum-doublet chemotherapy has been the standard first-line treatment for advanced non-small-cell lung cancer (NSCLC), but its prognosis is poor 2-4. In the recent decade, precision-targeted therapies for patients with oncogenic alterations, such as epidermal growth factor receptor (*EGFR*) and B-type Raf kinase V600E (*BRaf^V600E^)* mutations or anaplastic lymphoma kinase (*ALK*) and ROS proto-oncogene 1 (*ROS1*) fusions, have provided favorable effectiveness and survival benefits 5-7. Unfortunately, almost all patients ultimately acquire resistance to targeted therapies. Hence, novel treatment strategies are urgently needed.

Antibodies targeting the programmed death-ligand 1 (PD-L1)/programmed death-1 (PD-1) pathway represent an important advance in the management of metastatic NSCLC. In recent years, anti-PD-L1/anti-PD-1 immune checkpoint inhibitors (ICIs) have become the standard of care for previously treated NSCLC without a targetable oncogene, and have rapidly become first-line treatment for patients with advanced NSCLC 2, 3, 8-11.

Atezolizumab, an anti-PD-L1 antibody, has been shown to provide clinically relevant improvement of overall survival (OS) versus docetaxel in patients with previously treated metastatic NSCLC, regardless of PD-L1 expression 12. In addition, patients with *EGFR* mutation-positive disease also have similar overall survival benefit with atezolizumab and docetaxel 12. Atezolizumab has also shown a promising efficacy and an acceptable safety profile when combined with platinum-doublet chemotherapy in patients who have not previously received chemotherapy for NSCLC 13, 14. Even as a first-line treatment, atezolizumab monotherapy provides a significantly longer OS than platinum-based chemotherapy in patients with NSCLC with high PD-L1 expression, regardless of the histologic type 8. In the phase 3 IMpower150 study, the addition of atezolizumab to bevacizumab plus carboplatin and paclitaxel as the first-line treatment for nonsquamous metastatic NSCLC resulted in a significant improvement in progression-free survival (PFS) and OS 15. A survival benefit was observed across key subgroups, including those with varying levels of PD-L1 expression, with no new safety risks identified with the combination treatment.

Atezolizumab has been approved for patients with metastatic NSCLC in Taiwan since 2017. Despite its efficacy in clinical trials, little is known about real-world clinical outcomes of atezolizumab in patients with lung cancer, especially in the Asian population. Additionally, it remains unclear whether the choice of frontline treatment affects the effectiveness of subsequent atezolizumab treatment in patients with metastatic NSCLC. Especially, osimertinib has been reimbursed by the National Health Insurance (NHI) of Taiwan since April 2020. The purpose of this study was to investigate the impact of frontline treatments in patients who received subsequent atezolizumab-based regimens in real-world settings in Taiwan.

## Material and Methods

### Patients

This multicenter, observational, retrospective study was conducted at six medical centers in Taiwan. The participating institutions included three hospitals in northern Taiwan (National Taiwan University Hospital, Taipei Veterans General Hospital, and Chang Gung Memorial Hospital Linkou Branch), one in central Taiwan (China Medical University Hospital), and two in southern Taiwan (Chang Gung Memorial Hospital Kaohsiung Branch and Kaohsiung Medical University Chung-Ho Memorial Hospital). The study was approved by the Institutional Review Board of each participating medical center, which waived the requirement for informed consent.

The inclusion criteria included patients who had tumors which were histologically or cytologically confirmed diagnosis of advanced or metastatic lung cancers, and received at least one dose of atezolizumab before June 30, 2019. Exclusion criteria included patients participating the interventional clinical trials of atezolizumab, or patients whose follow-up duration after the first administration of atezolizumab was less than 4 weeks were excluded. Lung cancer histology was classified according to the World Health Organization classification of lung tumors 16. The lung cancer stage was determined according to the 8^th^ Edition of the International Association for the Study of Lung Cancer tumor-node-metastasis staging system 17. The patients had previously received at least one line of systemic therapy for unresectable, locally advanced, or metastatic NSCLC. Patients were included in the analysis irrespective of the PD-L1 status.

The clinical and demographic characteristics of the patients, treatment medications, and responses were recorded. The treatment medications included chemotherapy, TKI therapy, ICIs, or radiotherapy. Smoking history before lung cancer diagnosis, smoking duration, and the number of packs of cigarettes were recorded. Patients who had smoked <100 cigarettes in their lifetime were defined as nonsmokers, and all others were categorized as smokers 18.

### Response evaluation of patients with NSCLC

The treatment responses were evaluated and recorded according to the Response Evaluation Criteria in Solid Tumors guidelines (version 1.1), which included complete response (CR), partial response (PR), stable disease (SD), and progressive disease (PD) 19. PFS was defined as the period from the initiation of atezolizumab treatment to disease progression or death. OS was defined as the period from the date of initial atezolizumab treatment to the date of death.

### Statistical analysis

The SPSS software (version 26.0 for Mac; SPSS Inc., Chicago, IL, USA) was used for all statistical analyses. Categorical variables were analyzed using the chi-squared test. If the sampling variability was ≤ 5, Fisher's exact test was applied. Statistical significance was set at a two-sided *P*-value of < 0.05. The nonparametric Mann-Whitney *U*-test was used to compare the median ages between two groups.

The propensity score (PS) for the probability of atezolizumab monotherapy or combination therapy was created through a logistic regression model, which included potential confounders such as, sex, age, Eastern Cooperative Oncology Group performance status (ECOG PS), smoking, histology (adenocarcinoma vs. non-adenocarcinoma), brain metastasis, PD-L1 expression, line of atezolizumab, prior EGFR-TKI, prior platinum therapy, prior ICIs, prior pemetrexed, and prior osimertinib. A 1:1 matched cohort group of atezolizumab monotherapy and combination therapy was created. The survival outcome analysis of both original cohort and PSM cohort were performed.

The Kaplan-Meier method was used to plot survival curves, and the log-rank test was used for comparison between groups. The predictive factors of PFS and the potential prognostic factors of OS were evaluated by multivariate Cox regression model. The selection of possible predictors and prognostic factors were on the basis of previous studies investigating the prognostic factors of survival in lung cancer, especially for 2^nd^-line ICIs 20, 21, including: sex, smoking, performance status, tumor histology, brain metastasis, PD-L1 tumor proportion scores (TPS), *EGFR* mutation status, line of atezolizumab, atezolizumab monotherapy or combination therapy, prior treatments (EGFR-TKI, platinum therapy, ICIs, pemetrexed, and osimertinib).

## Results

### Patient distribution and baseline clinical characteristics

There were 160 patients who received atezolizumab, including 144 patients with NSCLC and 16 with small-cell lung cancer. Of the 144 patients with NSCLC, 16 received atezolizumab as the first-line treatment. 128 patients receiving atezolizumab as the second- or later-line (2L+) treatment were enrolled in this study. The median follow-up period of the 128 patients were 41.2 (95% confidence interval [CI]: 37.6-45.8) months.

The 128 patients had a median age of 60.8 (range: 32.8-83.2) years (Table 1). Sixty-four (50.0%) patients were females, and 75 (58.6%) were nonsmokers. The tumor histology included 97 (75.8%) adenocarcinoma. Twenty-four patients (18.8%) had tumor with high PD-L1 TPS (≥50%). The *EGFR* mutation status were 42 (32.8%) *EGFR* mutations, 66 (51.6%) wild type of *EGFR*, and 20 (15.6%) no recording data.

Thirty-eight (29.7%) patients were treated with atezolizumab as the second-line treatment, and 90 (70.3%) patients received atezolizumab as the third- or subsequent-line treatment. The median number of prior lines of systemic treatment was 4 (range: 2-11). A total of 57 (44.5%) patients received atezolizumab monotherapy, and 71 (55.5%) patients received atezolizumab combination therapy, including 44 (62.0%; 44 of 71) dual therapy, 20 (28.2%) triple therapy and 7 (9.9%) quadruple therapy (Supplementary Table S1). Before atezolizumab-containing treatment, there were 13 patients treated with ICIs (Supplementary Table S2), 62 patients with EGFR-TKIs, 110 patients with platinum-based chemotherapy, and 85 patients with pemetrexed.

### Treatment responses and survival analysis in patients with atezolizumab as 2L+ atezolizumab-containing treatment

Among the 128 patients with NSCLC who received 2L+ atezolizumab, the maximum response to atezolizumab treatment was CR in 1 patient (0.8%), PR in 12 patients (9.4%), SD in 37 patients (28.9%), and PD in 78 patients (60.9%). The response rate was 10.2%. The median PFS was 3.5 (95% CI: 2.9-4.1) months, and median OS was 10.7 (95% CI: 9.4-12.0) months.

PFS was used to evaluate the potential predictive factor and the impact of front-line treatments before atezolizumab-containing treatment (Table 2). There was no significant difference in mPFS between patients with different PD-L1 TPS (<50% versus ≥50% *versus* No data: 3.2 months *versus* 3.1 months *versus* 3.8 months; *p* = 0.724) (Supplementary Figure S1A). There was also no significant difference in mPFS between the patients with *EGFR* mutations (3.2 months), wild type of *EGFR* (3.4 months) and without *EGFR* mutation data (3.9 months; *p* = 0.344) (Supplementary Figure S1B). Thirteen patients who had received ICIs before atezolizumab-containing treatment had a shorter mPFS (2.4 months *versus* 3.5 months; *p* = 0.009) than those without prior ICIs (Supplementary Figure S2A). There was no significant difference in mPFS between patients with and without prior EGFR-TKI (3.5 months *versus* 3.3 months; *p* = 0.341).

**Figure 1:**
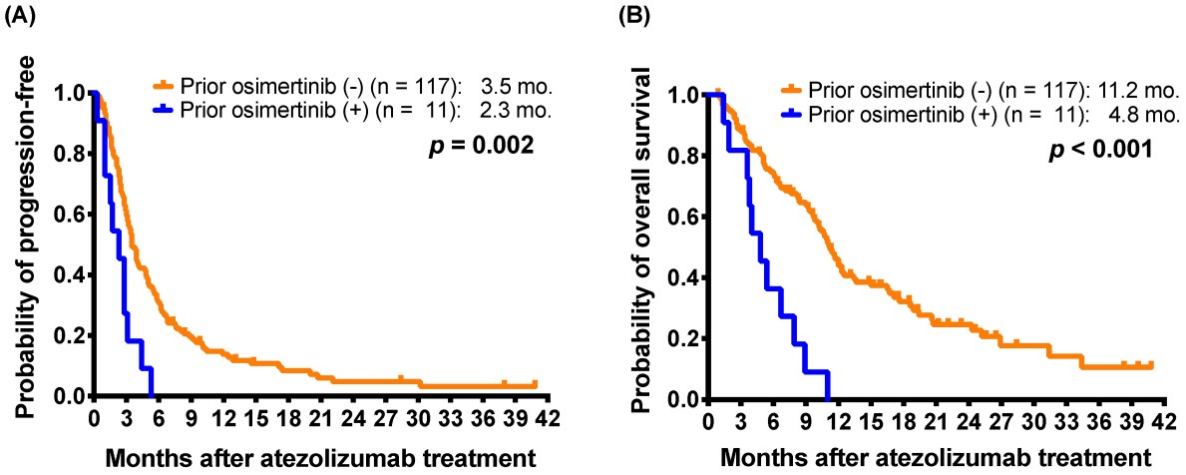
Kaplan-Meier estimates of **(A)** progression-free survival and **(B)** overall survival of patients with and without osimertinib before atezolizumab treatment (log-rank test) in the patients with atezolizumab as 2L+ atezolizumab-containing treatment.

Furthermore, 11 patients had received osimertinib treatment before atezolizumab-containing treatment. The 11 patients received osimertinib after acquiring resistance to EGFR-TKI therapy, including gefitinib, erlotinib, and afatinib (seven, two, and two patients, respectively). All of the patients were nonsmokers and received atezolizumab in the third- or subsequent-line settings (range: 4-10, median: 6). The 11 patients who had received osimertinib treatment before atezolizumab had a shorter mPFS than those who had not received prior osimertinib (2.3 months versus 3.5 months; *p* = 0.002) (Figure 1A).

For mOS, the patients who received EGFR TKIs before atezolizumab had shorter mOS than those who had not received prior EGFR TKI (10.1 months *versus* 12.0 months; *p* = 0.048) (Table 3). Furthermore, the patients who received osimertinib before atezolizumab also had shorter mOS than those who had not received prior osimertinib (4.8 months *versus* 11.2 months; *p* < 0.001) (Figure 1B). The patients who had received ICIs before an atezolizumab-containing regimen had a shorter mOS (6.3 *versus* 11.0 months; *p* = 0.030) than those without prior ICI treatment (Supplementary Figure S2B). In addition, patients with better performance status (ECOG PS 0-1) (11.7 months *versus* 5.7 months; *p* < 0.001), patients with adenocarcinoma histology (11.1 months versus 6.6 months; *p* = 0.021) had longer mOS.

**Figure 2:**
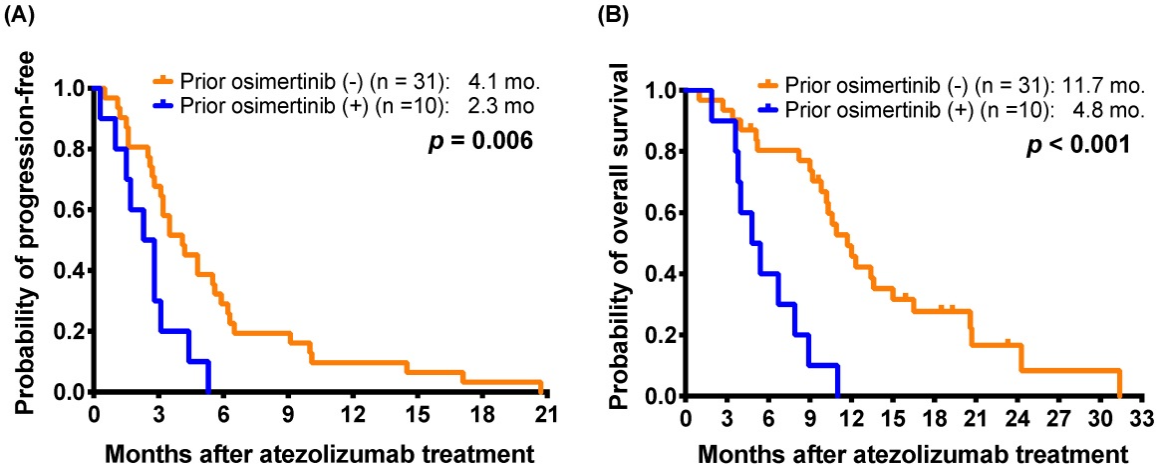
For 41 patients harboring non-squamous NSCLC with *EGFR* mutations, Kaplan-Meier estimates of **(A)** progression-free survival and **(B)** overall survival of patients with and without osimertinib exposure before atezolizumab treatment (log-rank test).

To more clarify the impact of osimertinib on the effectiveness of subsequent atezolizumab treatment, we extracted a subgroup including 41 patients harboring nonsquamous NSCLC with *EGFR* mutations (Supplementary Table S3). After exclusion of one osimertinib-treated patient without *EGFR* mutation data, the subgroup enrolled 10 patients who had received osimertinib after acquired resistance to first- or second-generation EGFR-TKIs and before atezolizumab-containing treatment. The difference in the response rates between the patients with and without osimertinib treatment before atezolizumab therapy did not reach statistical significance (0.0% [0/10] vs. 9.7% [3/31], *p* = 0.564, by Fisher's exact test). The patients who received osimertinib before atezolizumab had a shorter mPFS (2.3 months versus 4.1 months; *p* = 0.006) and a mOS (4.8 months versus 11.7 months; *p* < 0.001) than those who had not received prior osimertinib (Figure 2A and 2B).

### Predictive and prognostic factors in patients with atezolizumab as 2L+ atezolizumab-containing treatment

Multivariate analysis was performed using the Cox regression model to determine potential predictive factors of PFS (Table 2). Patients who had received osimertinib before atezolizumab-containing treatment had significantly shorter PFS than those who did not receive prior Osimertinib (HR, 2.94; 95% CI, 1.34-6.47; *p* = 0.007). In addition, prior ICIs (HR, 3.03; 95% CI, 1.54-5.95; *p* = 0.001) was significantly associated with shorter PFS.

For OS, multivariate analysis was carried out to identify potential prognostic factors for survival and showed that prior osimertinib treatment (HR: 3.55; 95% CI, 1.57-8.03; *p* = 0.002), prior ICI treatment (HR: 3.18; 95% CI, 1.53-6.61; *p* = 0.002) and a poor performance status (HR: 2.70; 95% CI, 1.46-4.97; *p* = 0.001) were associated with shorter OS. Meanwhile, adenocarcinoma histology was a favorable prognostic factor (HR: 0.31; 95% CI, 0.16-0.60; *p* < 0.001) (Table 3).

### Comparing treatment efficacy and survival of matched monotherapy and combination therapy of atezolizumab

To compare the clinical efficacy between atezolizumab monotherapy and combination therapy, we conducted a propensity-scored 1:1 matched cohort, and there were total of 34 patient pairs (PSM cohort) who received monotherapy/combination therapy of atezolizumab from the original cohort. The demographic and clinical characteristics were balanced between the matched groups (Table 4).

**Figure 3:**
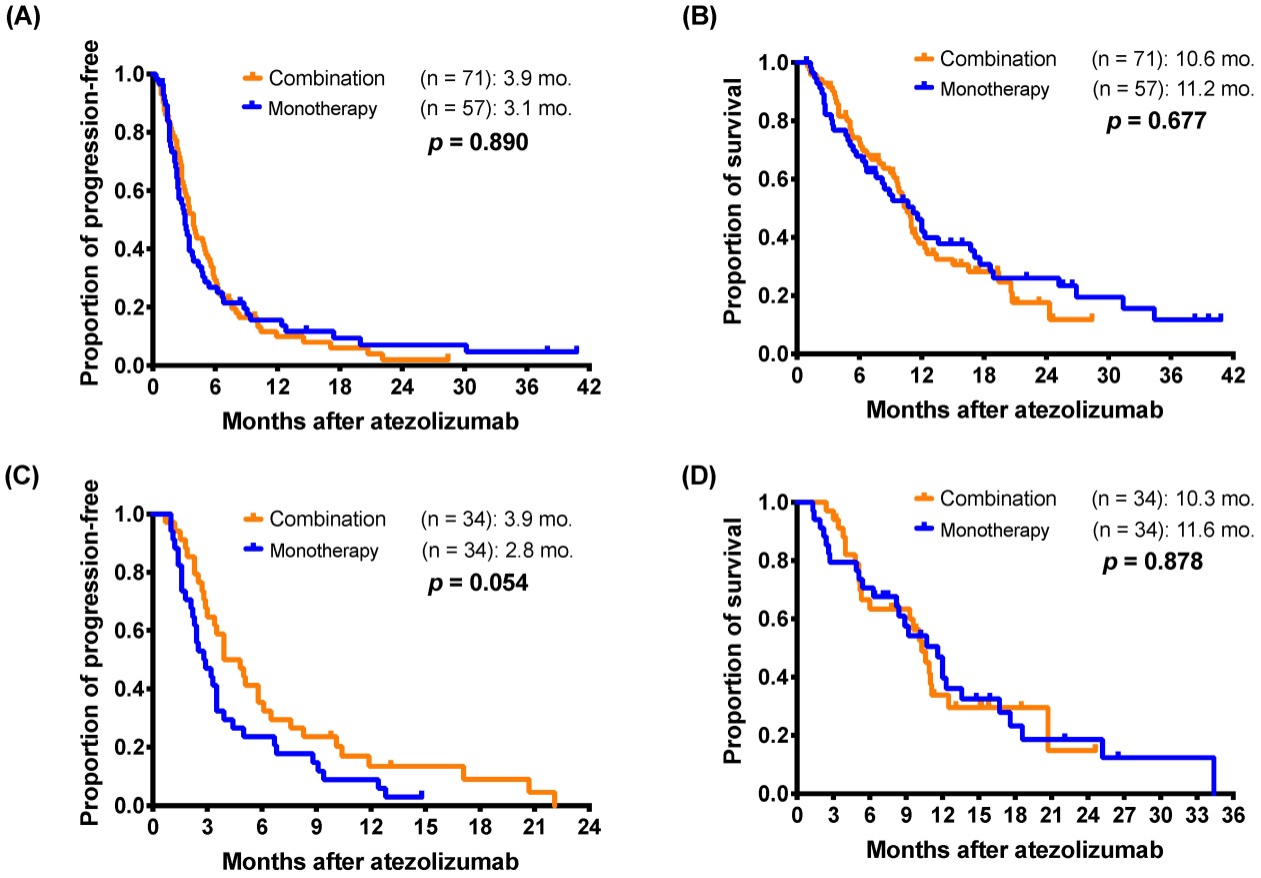
Kaplan-Meier estimates of **(A)** progression-free survival and **(B)** overall survival of patients who received monotherapy or combination therapy of atezolizumab (log-rank test) in the original cohort. For propensity-score matching cohort, **(C)** progression-free survival and **(D)** overall survival of patients who received monotherapy or combination therapy.

In the PSM cohort, the response rate were 2.9% (1 of 34) in monotherapy group and 11.8% (4 of 34) in the combination therapy group (*p* = 0.356, by Fisher's exact test). In the original cohort, there were no significant differences in mPFS (3.1 months *versus* 3.9 months; *p* = 0.890) and mOS (11.2 months *versus* 10.6 months; *p* = 0.677) between monotherapy group and combination therapy group (Figure 3A and 3B). Pertaining to the PSM cohort, the results of outcomes analysis were highly consistent with the original cohort. There were also no significant differences in mPFS (2.8 months *versus* 3.9 months; *p* = 0.054) and mOS (11.6 months *versus* 10.3 months; *p* = 0.878) between patients who received monotherapy and combination therapy of atezolizumab (Figure 3C and 3D).

In addition, multivariate analysis for PFS and OS of the PSM cohort also supported the results of the original cohort (Supplementary Table S4). Patients who had received osimertinib before atezolizumab-containing treatment had significantly shorter PFS (HR, 2.73; 95% CI, 1.05-7.07; *p* = 0.039) and OS (HR: 3.30; 95% CI, 1.16-9.41; *p* = 0.026) than those who did not receive prior osimertinib. Prior ICI treatment (HR: 3.17; 95% CI, 1.19-8.45; *p* = 0.021) and a poor performance status (HR: 3.26; 95% CI, 1.55-6.86; *p* = 0.002) were also associated with shorter OS.

## Discussion

This multicenter observational study explored the clinical prognostic factors in patients with NSCLC who received atezolizumab-containing regimens as a 2L+ treatment in real-world settings in Taiwan. Patients who had received osimertinib treatment before atezolizumab experienced a shorter mPFS and mOS than those who had not received prior osimertinib. Moreover, poor performance status and prior ICIs exposure before atezolizumab treatment were associated with a poor prognosis. Given the reported impacts of prior osimertinib treatment in patients who received subsequent ICIs, this is an important issue for future studies.

Osimertinib has been approved by the FDA as a frontline treatment in patients with *EGFR*-mutant, metastatic NSCLC. In Taiwan, osimertinib has not been reimbursed until April 2020. However, it is unknown whether osimertinib impacts the effectiveness of subsequent treatments, especially ICIs. The current study showed that patients with prior osimertinib treatment had a shorter mPFS than those without osimertinib treatment. This finding was consistent with the post-hoc analysis results of a phase 2 trial, which showed that patients with acquired resistance to osimertinib had a shorter PFS with atezolizumab combination therapy than those without osimertinib exposure 22. Furthermore, the current study showed that prior osimertinib treatment was a poor prognosis factor, likely because patients exposed to osimertinib represented a subgroup that had exhausted multiple lines of TKI therapy and/or potentially acquired resistance *EGFR* mutations, such as T790M. This observation may be hinted by the results of the phase 3 KEYNOTE-789 trial (NCT03515837), which evaluates the efficacy and safety of pemetrexed plus platinum chemotherapy (carboplatin or cisplatin), with or without pembrolizumab, in the treatment of adults with EGFR-TKI-resistant, *EGFR*-mutated, metastatic nonsquamous NSCLC tumors, including first-line osimertinib failure.

Recently, clinical trials of frontline immunotherapy demonstrated disappointing results for patients with NSCLC with *EGFR* or *ALK* alterations 23-25. Several double-blind, randomized controlled clinical trials have shown poor second-line treatment outcomes for *EGFR*-mutant NSCLC treated with a single ICI agent 12, 26, 27. In addition to treatment effectiveness, safety issues should be considered for concurrent or sequential immunotherapy and targeted therapy. Patients who received a combination of immunotherapy and EGFR-TKIs demonstrated more toxicities, including pyrexia, pneumonitis, and abnormal liver function 28, 29. Furthermore, osimertinib plus durvalumab treatment was terminated early in the TATTON trial owing to increased reporting of interstitial lung disease 30. Lisberg *et al.* reported treatment of seven patients with PD-L1-positive, *EGFR*-mutant, advanced NSCLC with pembrolizumab before EGFR-TKI therapy 25. Apart from treatment futility, one patient developed fatal pneumonitis on erlotinib, and another patient died. Although these adverse effects may have resulted from an EGFR-TKI, their increased occurrence with pembrolizumab is concerning 31. Sequential ICI and ALK-TKI (crizotinib) treatment was associated with a significantly increased risk of hepatotoxicity in patients with *ALK*, *ROS1*, or MNNG HOS transforming gene (*MET*) exon 14 alterations 32. Thus, it is important to arrange the combination or sequence of targeted therapy and ICIs during the entire treatment course of NSCLC.

A randomized phase 3 study, the OAK trial (NCT02008227), revealed that atezolizumab improved OS over that with docetaxel in patients with locally advanced or metastatic NSCLC who had previously received one to two lines of chemotherapy, including at least one platinum-based treatment (HR: 0.75; 95% CI, 0.64-0.89; *p* = 0.0006) 33, and the updated response rate (RR), PFS, and OS were 14.6%, 2.8 months, and 13.8 months, respectively 33. In addition, the global phase III/IV TAIL study showed an RR of 11.1%, mPFS of 2.7 months, and mOS of 11.1 months 34. The current study in real-world settings showed a RR of 10.2%, mPFS of 3.5 months, and mOS of 10.7 months, which was similar to the results of the clinical trial 33. However, the proportions of patients with *EGFR* mutation-positive status were 10% in the OAK trial and 4% in the TAIL trial 12, 34, which were significantly lower than the current study (32.8%). The OAK trial showed that *EGFR* mutation status did not impact on the efficacy of atezolizumab as 2^nd^-line treatment, and it is similar to the current studies. Furthermore, the above two clinical trials did not explore the impact of osimertinib exposure on the treatment effectiveness of atezolizumab. The current study showed that osimertinib exposure before atezolizumab was associated with shorter mPFS and mOS.

PD-L1 is currently widely validated and accepted as a biomarker of response to ICIs 35. Although atezolizumab as first-line monotherapy showed clinical survival benefit in patients with NSCLC with high PD-L1 expression 8, the OAK trial revealed that patients with previously treated metastatic NSCLC have survival benefit from atezolizumab treatment regardless of PD-L1 expression, histology, or *EGFR* mutation status 12. The current study also showed the same results that neither PD-L1 expression nor *EGFR* mutation status were associated with PFS or OS. The mechanism of the different response in NSCLC patients with high- or low-expression PD-L1 when treating with atezolizumab as first-line or second-line is unclear.

Whether immunotherapy has a potential role in patients with driver mutations remains debated. Most of the ICI clinical trials excluded patients with *EGFR* mutations or *ALK* fusions because the response rate to ICI monotherapy was low for NSCLC with *EGFR* mutations 12, 23. Cohorts E and F in the phase 1/2 KEYNOTE-021 study (NCT02039674) also showed that pembrolizumab plus gefitinib or erlotinib was not a feasible treatment option because of grade 3/4 liver toxicity or no improvement in the response rate, respectively 36. However, the phase 3 IMpower150 trial (NCT02366143) demonstrated that the four-drug combination of atezolizumab, bevacizumab, carboplatin, and paclitaxel, when used as the frontline treatment for patients with metastatic nonsquamous NSCLC, improved OS (HR: 0.78; 95% CI, 0.64-0.96; *p* = 0.02) and PFS (HR: 0.62; 95% CI, 0.52-0.74; *p* < 0.001) vs. bevacizumab plus carboplatin/paclitaxel 15. Notably, a survival benefit was also observed in patients with *EGFR* mutations who had received prior treatment with EGFR-TKIs 37. In 2020, Lam *et al.* reported the results of a phase 2 trial in which atezolizumab/bevacizumab/pemetrexed/carboplatin provided a response rate of over 60% and an mPFS of 9.4 months in patients with TKI-resistant, *EGFR*-mutant, metastatic lung cancer 22. More clinical trials are necessary to clarify whether atezolizumab combination therapy may be effective in patients with acquired resistance to EGFR-TKIs.

Although the U.S. Food and Drug Administration (FDA) has approved ICIs as a standard of care for previously treated patients with NSCLC, it remains unclear whether the frontline medications affect the effectiveness of subsequent immunotherapy in these patients. Limited data are available regarding the prognosis of patients who receive immunotherapy in the frontline setting prior to atezolizumab treatment. Hernando-Calvo *et al.* reported that prior ICIs were not associated with the best treatment responses to subsequent ICIs, and only metastatic burden was a significant predictor of PFS by multivariate analysis 38. Although our data also showed that there was no significant difference in the response rates to atezolizumab between patients with and without prior ICI treatment, the former group had shorter PFS and OS. The difference may be due to the fact that the previous study enrolled patients with various tumor types, including melanoma (45%), NSCLC (21%), and head and neck squamous cell carcinoma 38. Further, different ICIs may have different treatment effects.

Variable laboratory biomarkers have been associated with treatment efficacy or prognosis of immunotherapy 39-43. Mezquita et al. reported that pretreatment lung immune prognostic index (LIPI), combining derived neutrophils/(leukocytes minus neutrophils) ratio (dNLR) greater than 3 and LDH greater than upper limit of normal (ULN), was correlated with worse outcomes for ICI 42. Zho et al. showed that circulating immune cell ratio and tumor markers could be as the potential predict factors of atezolizumab for lung cancer patients 40. In addition, higher neutrophil to lymphocyte ratio (NLR), higher ALP, increasing white blood cell counts, and abnormally low albumin and low chloride levels associated with poor prognosis 44-47. Although identification of biomarkers for patients who had favorable response to ICIs is urgent and important, most of these trials were retrospectively designed or had small case numbers. Further prospective randomized controlled trials and unified companion diagnostic devices are necessary to validate these candidate biomarkers. Although we did not enrolled the serum biomarkers as prognosis factors due to heterogenicity background of the enrolled patients, the results of the current study may serve as the basis for conducting future prospective studies using these factors to identify the patients who had directly benefit from atezolizumab treatment.

This study had some limitations. First, we retrospectively extracted patient-level data, and the nature of the retrospective study design may not completely exclude inherent biases. The atezolizumab-containing treatments were heterogeneous, and the results of this study may have limited generalizability to all patients. Second, ICIs and osimertinib has not been reimbursed by NIH of Taiwan until early 2020, and ICIs reimbursement criteria excluded patients with *EGFR* mutations. So, the number of osimertinib-treated patients before atezolizumab was less during the enrollment period of the study. Nonetheless, the enrollment of a large population from the six medical centers across the country and the nationwide data provided valuable information for future clinical decision making. Future randomized controlled designs will help strengthen the data of the hypothesis-generating research. Third, we did not collect the delicate subsequent therapies after disease progression to atezolizumab treatment, and it may have impact on OS.

In conclusion, prior osimertinib exposure was associated with poor clinical outcomes of subsequent atezolizumab-containing treatment of NSCLC. Additional studies with larger sample sizes are required to validate these findings.

## Supplementary Material

Supplementary figures and tables.

## Figures and Tables

**Table 1:** Clinical characteristics of NSCLC patients who had received atezolizumab as the second- or later-line (2L+) treatment

Factor	Patients (%)
Total patients, n (%)	128 (100.0%)
Age (median, years) (range)	60.8 (32.8-83.2)
**Sex**	
Female	64 (50.0%)
Male	64 (50.0%)
**Smoking status**	
Nonsmokers	75 (58.6%)
Smokers	53 (41.4%)
**ECOG PS**	
0-1	103 (80.5%)
≥2	25 (19.5%)
**Histology**	
Non-adenocarcinoma	31 (24.2%)
Adenocarcinoma	97 (75.8%)
**Brain Metastasis**	
No	78 (60.9%)
Yes	50 (39.1%)
**PD-L1 TPS**	
<50%	58 (45.3%)
≥50%	24 (18.8%)
No data	46 (35.9%)
***EGFR***	
Wild type	66 (51.6%)
Mutant	42 (32.8%)
No data	20 (15.6%)
**Line of atezolizumab**	
Second	38 (29.7%)
≥Third	90 (70.3%)
**Atezolizumab therapy**	
Monotherapy	57 (44.5%)
Combination	71 (55.5%)
**Prior medications**	
EGFR-TKI	62 (48.4%)
ICIs	13 (10.2%)
Platinum	110 (85.9%)
Pemetrexed	85 (66.4%)

*EGFR*, epidermal growth factor receptor gene; EGFR-TKI, epidermal growth factor receptor tyrosine kinase inhibitor; ECOG PS, Eastern Cooperative Oncology Group performance status; TPS, tumor proportion score; ICIs, immune checkpoint inhibitors.

**Table 2:** Multivariate analysis of predictive factors for PFS in patients who received atezolizumab as a second- or subsequent-line treatment

Factor	Number of patients	PFS (months)	Univariate analysis	Multivariate analysis	
			*P*	HR (95% CI)	*P*
**Sex**					
Female	64	3.5		1	
Male	64	3.4	0.688	1.46 (0.80-2.64)	0.215
**Smoking history**					
Nonsmokers	75	3.5		1	
Smokers	53	3.5	0.744	1.07 (0.59-1.93)	0.832
**ECOG PS**					
0-1	103	3.5		1	
≥2	25	3.5	0.249	1.13 (0.66-1.93)	0.651
**Histology**					
Non-adenocarcinoma	31	3.5		1	
Adenocarcinoma	97	3.5	0.246	0.58 (0.31-1.08)	0.087
**Brain metastasis**					
No	78	3.9		1	
Yes	50	2.9	0.131	1.41 (0.91-2.30)	0.125
**PD-L1 TPS**					
<50%	58	3.2		1	
≥50%	24	3.1		1.29 (0.72-2.31)	0.398
No data	46	3.8	0.724	1.09 (0.69-1.70)	0.724
***EGFR***					
Wild type	66	3.4		1	
Mutant	42	3.2		0.82 (0.42-1.58)	0.544
No data	20	3.9	0.344	1.22 (0.66-2.24)	0.528
**Line of atezolizumab**					
second	38	3.5		1	
≥third	90	3.2	0.820	1.24 (0.76-2.03)	0.395
**Atezolizumab therapy**					
Monotherapy	57	3.1		1	
Combination	71	3.9	0.890	0.86 (0.55-1.35)	0.503
**Prior EGFR-TKI**					
No	66	3.3		1	
Yes	62	3.5	0.463	1.19 (0.66-2.15)	0.570
**Prior platinum therapy**					
No	18	3.3		1	
Yes	110	3.5	0.341	0.56 (0.29-1.12)	0.100
**Prior immunotherapy**					
No	115	3.5		1	
Yes	13	2.4	0.009	3.03 (1.54-5.95)	0.001
**Prior pemetrexed**					
No	43	3.9		1	
Yes	85	3.1	0.886	1.22 (0.69-2.17)	0.496
**Prior osimertinib**					
No	117	3.5		1	
Yes	11	2.3	0.002	2.94 (1.34-6.47)	0.007

PFS, progression-free survival; HR, hazards ratio; CI, confidence interval; ECOG PS, Eastern Cooperative Oncology Group performance status; PD-L1, programmed death-ligand 1; TPS, tumor proportion score; *EGFR*, epidermal growth factor receptor gene; EGFR-TKI, epidermal growth factor receptor tyrosine kinase inhibitor.

**Table 3:** Multivariate analysis of prognostic factors for OS in patients who received atezolizumab as a second- or subsequent-line treatment

Factor	Number of patients	OS (months)	Univariate analysis	Multivariate analysis	
			*P*	HR (95% CI)	*P*
**Sex**					
Female	64	10.3		1	
Male	64	11.1	0.977	0.73 (0.39-1.37)	0.329
**Smoking history**					
Nonsmokers	75	10.1		1	
Smokers	53	11.6	0.707	0.94 (0.47-1.88)	0.871
**ECOG PS**					
0-1	103	11.7		1	
≥2	25	5.7	<0.001	2.70 (1.46-4.97)	0.001
**Histology**					
Non-adenocarcinoma	31	6.6		1	
Adenocarcinoma	97	11.1	0.021	0.31 (0.16-0.60)	<0.001
**Brain metastasis**					
No	78	10.7		1	
Yes	50	10.3	0.563	0.82 (0.49-1.36)	0.442
**PD-L1 TPS**					
<50%	58	10.2		1	
≥50%	24	7.6		1.84 (0.96-3.54)	0.069
No data	46	11.7	0.341	1.10 (0.65-1.88)	0.728
***EGFR***					
Wild type	66	11.0		1	
Mutant	42	10.2		0.65 (0.29-1.47)	0.299
No data	20	11.4	0.241	1.53 (0.78-3.01)	0.221
**Line of atezolizumab**					
second	38	11.2		1	
≥third	90	10.2	0.630	1.58 (0.88-2.84)	0.129
**Atezolizumab therapy**					
Monotherapy	57	11.2		1	
Combination	71	10.6	0.677	0.85 (0.50-1.44)	0.541
**Prior EGFR-TKI**					
No	66	12.0		1	
Yes	62	10.1	0.048	1.40 (0.73-2.70)	0.317
**Prior platinum therapy**					
No	18	8.3		1	
Yes	110	10.9	0.115	0.68 (0.30-1.54)	0.351
**Prior immunotherapy**					
No	115	11.0		1	
Yes	13	6.3	0.030	3.18 (1.53-6.61)	0.002
**Prior pemetrexed**					
No	43	11.2		1	
Yes	85	10.3	0.473	1.08 (0.57-2.06)	0.819
**Prior osimertinib**					
No	117	11.2		1	
Yes	11	4.8	<0.001	3.55 (1.57-8.03)	0.002

OS, overall survival; HR, hazards ratio; CI, confidence interval; ECOG PS, Eastern Cooperative Oncology Group performance status; PD-L1, programmed death-ligand 1; TPS, tumor proportion score; *EGFR*, epidermal growth factor receptor gene; EGFR-TKI, epidermal growth factor receptor tyrosine kinase inhibitor.

**Table 4:** Clinical characteristics of patients who had received monotherapy or combination therapy of atezolizumab

Factor	Original cohort of atezolizumab		*P*	Propensity-score matching cohort of atezolizumab		*P*
	Monotherapy	Combination		Monotherapy	Combination	
Total patients, n (%)	57 (44.5%)	71 (55.5%)		34 (50.0%)	34 (50.0%)	
Age (median, years) (range)	62.0 (32.8-80.2)	58.5 (35.1-83.2)	0.294^§^	62.6 (42.5-80.2)	61.6 (36.1-83.2)	0.536^§^
**Sex**			0.594			0.808
Female	30 (52.6%)	34 (47.9%)		16 (47.1%)	15 (44.1%)	
Male	27 (47.4%)	37 (52.1%)		18 (52.9%)	19 (55.9%)	
**Smoking status**			0.112			1.000
Nonsmokers	29 (50.9%)	46 (64.8%)		18 (52.9%)	18 (52.9%)	
Smokers	28 (49.1%)	25 (35.2%)		16 (47.1%)	16 (47.1%)	
**ECOG PS**			0.611			0.752*
0-1	47 (82.5%)	56 (78.9%)		29 (85.3%)	27 (79.4%)	
≥2	10 (17.5%)	15 (21.1%)		5 (14.7%)	7 (20.6%)	
**Histology**			0.031			0.770
Non-adenocarcinoma	19 (33.3%)	12 (16.9%)		8 (23.5%)	7 (20.6%)	
Adenocarcinoma	38 (66.7%)	59 (83.1%)		26 (76.5%)	27 (79.4%)	
**Brain metastasis**			0.055			1.000
No	40 (70.2%)	38 (53.5%)		23 (67.6%)	23 (67.6%)	
Yes	17 (29.8%)	33 (46.5%)		11 (32.4%)	11 (32.4%)	
**PD-L1 TPS**			0.159			0.275
<50%	21 (36.8%)	37 (52.1%)		17 (50.0%)	11 (32.4%)	
≥50%	14 (24.6%)	10 (14.1%)		5 (14.7%)	9 (26.5%)	
No data	22 (38.6%)	24 (33.8%)		12 (35.3%)	14 (41.2%)	
***EGFR* mutations**			0.007			0.592
Wild type	33 (57.9%)	33 (46.5%)		18 (52.9%)	21 (61.8%)	
Mutant	11 (19.3%)	31 (43.7%)		9 (26.5%)	9 (26.5%)	
No data	13 (22.8%)	7 (9.9%)		7 (20.6%)	4 (11.8%)	
**Line of atezolizumab**			0.675			0.604
Second	18 (31.6%)	20 (28.2%)		10 (29.4%)	12 (35.3%)	
≥Third	39 (68.4%)	51 (71.8%)		24 (70.6%)	22 (64.7%)	
**Prior medications**						
EGFR-TKIs	16 (28.1%)	46 (64.8%)	<0.001	16 (47.1%)	16 (47.1%)	1.000
ICIs	4 (7.0%)	9 (12.7%)	0.383*	4 (11.8%)	1 (2.9%)	0.356*
Platinum	49 (86.0%)	61 (85.9%)	0.994	30 (88.2%)	29 (85.3%)	1.000*
Pemetrexed	35 (61.4%)	50 (70.4%)	0.283	24 (70.6%)	24 (70.6%)	1.000
osimertinib	3 (5.3%)	8 (6.3%)	0.344*	3 (8.8%)	2 (5.9%)	1.000*

*Fisher's exact test; ^§^Mann-Whitney *U*-test; ECOG PS, Eastern Cooperative Oncology Group performance status; TPS, tumor proportion score; TKI, tyrosine kinase inhibitor; ICIs, immune checkpoint inhibitors; *EGFR*, epidermal growth factor receptor gene; EGFR-TKI, epidermal growth factor receptor tyrosine kinase inhibitor.
