# Supplementary material for: Clinical outcomes of Atezolizumab Therapy for Previously-Treated Advanced-Stage Non-Small Cell Lung Cancer: A Real-World Study in Taiwan
Source: J Cancer. 2022 Jul 18;13(9):2922–32. doi: 10.7150/jca.74617 (PMC9330461; doi:10.7150/jca.74617)
Supplement: Supplementary file 1 — Supplementary figures and tables. [file jcav13p2922s1.pdf]

Supplemental Fig. 1. Kaplan–Meier survival curve of progression-free survival for patients with atezolizumab as 2L+ atezolizumab-containing treatment. There were no significant difference in PFS between patients with different (A) PD-L1 TPS (<50% *versus* ≥50% *versus* No data)(p = 0.724), or (B) *EGFR* mutation status (mutation *versus* wild type *versus* No data)(p = 0.344).

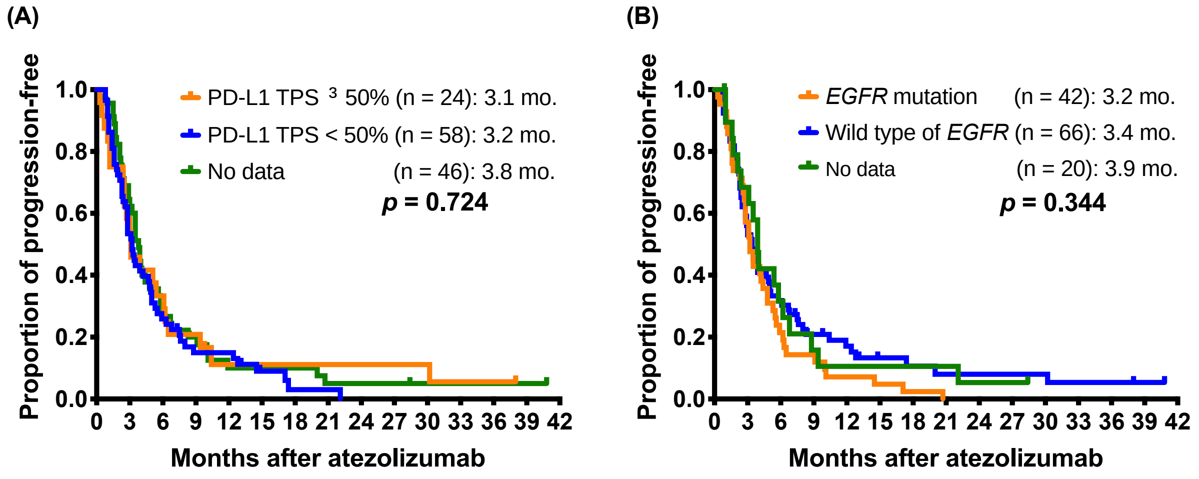

Figure 2. Kaplan–Meier estimates of (A) progression-free survival and (B) overall survival of patients with and without immunotherapy before atezolizumab treatment (log-rank test).

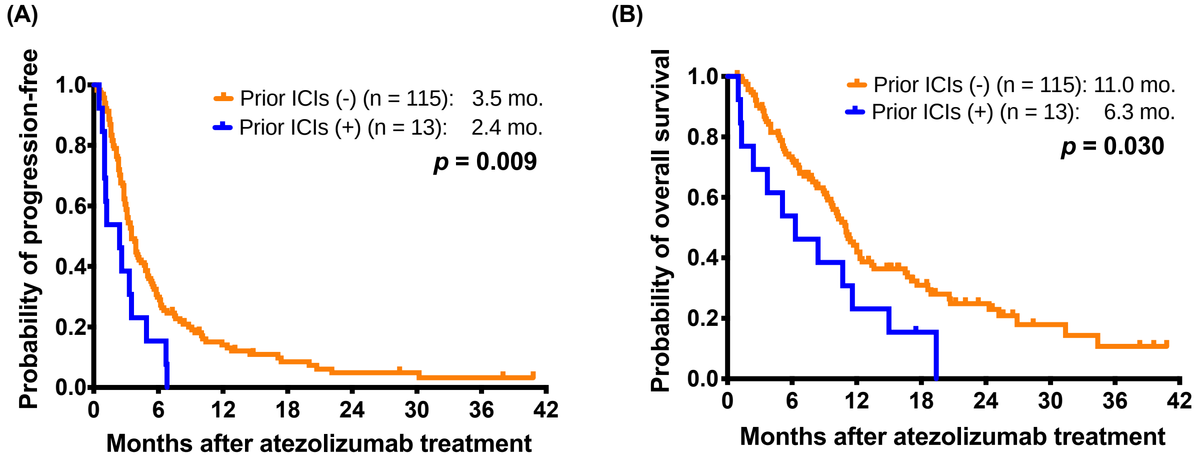

Supplemental Table S1 The different atezolizumab-containing regimens.

Treatment Regimens	Monotherapy (n = 57)	Combination therapy (n =71)
**Mono therapy**	**57(100.0%)**	**0(0.0%)**
Atezolizumab	57(100.0%)	0(0.0)
**Dual therapy**	**0(0.0%)**	**44(62.0%)**
Atezolizumab+Docetaxel	0(0.0%)	14(19.7%)
Atezolizumab+Gemcitabine	0(0.0%)	7(9.9%)
Atezolizumab+Vinorelbine	0(0.0%)	7(9.9%)
Atezolizumab+Pemetrexed	0(0.0%)	6(8.5%)
Atezolizumab+Paclitaxel	0(0.0%)	3(4.2%)
Atezolizumab+Bevacizumab	0(0.0%)	3(4.2%)
Atezolizumab+Ramucirumab	0(0.0%)	1(1.4%)
Atezolizumab+Afatinib	0(0.0%)	1(1.4%)
Atezolizumab+Alectinib	0(0.0%)	1(1.4%)
Atezolizumab+Ipilizumab	0(0.0%)	1(1.4%)
**Triple Therapy**	**0(0.0%)**	**20(28.2%)**
Atezolizumab+Pemetrexed+Cisplatin	0(0.0%)	5(7.0%)
Atezolizumab+Pemetrexed+Carboplatin	0(0.0%)	2(2.8%)
Atezolizumab+Paclitaxel+Cisplatin	0(0.0%)	2(2.8%)
Atezolizumab+Etoposide+Cisplatin	0(0.0%)	2(2.8%)
Atezolizumab+Gemcitabine+Cisplatin	0(0.0%)	1(1.4%)
Atezolizumab+Gemcitabine+Carboplatin	0(0.0%)	1(1.4%)
Atezolizumab+Cisplatin+Docetaxel	0(0.0%)	1(1.4%)
Atezolizumab+Cisplatin+Vinorelbine	0(0.0%)	1(1.4%)
Atezolizumab+Bevacizumab+Vinorelbine	0(0.0%)	3(4.2%)
Atezolizumab+Bevacizumab+Gemcitabine	0(0.0%)	1(1.4%)
Atezolizumab+Bevacizumab+Docetaxel	0(0.0%)	1(1.4%)
**Quadruple**	**0(0.0%)**	**7(9.9%)**
Atezolizumab+Bevacizumab+Pemetrexed+Cisplatin	0(0.0%)	2(2.8%)
Atezolizumab+Bevacizumab+Pemetrexed+Carboplatin	0(0.0%)	2(2.8%)
Atezolizumab+Bevacizumab+Paclitaxel+Carboplatin	0(0.0%)	1(1.4%)
Atezolizumab+Bevacizumab+Docetaxel+Carboplatin	0(0.0%)	1(1.4%)
Atezolizumab+Bevacizumab+Vinorelbine+Carboplatin	0(0.0%)	1(1.4%)

Supplementary Table S2. The different immune checkpoint inhibitor-containing regimens before atezolizumab-containing regimens.

Treatment Regimens	Number
**Mono therapy**	**5**
Pembrolizumab	3
Nivolumab	1
Spartalizumab	1
**Combination therapy**	**8**
Pembrolizumab+Paclitaxel	1
Pembrolizumab+Bevacizumab	1
Pembrolizumab+Pemetrexed+Carboplatin	1
Pembrolizumab+Paclitaxel+Cisplatin	1
Pembrolizumab+Paclitaxel+Carboplatin	1
Pembrolizumab+Bevacizumab+Carboplatin+Pemetrexed	1
Nivolumab+Erlotinib	1
Nivolumab+ Pemetrexed+Cisplatin+Afatinib	1

Supplementary Table S3 Clinical characteristics of *EGFR* mutation-positive patients who had received EGFR-TKIs before atezolizumab.

Factor	All patients	Osimertinib exposure before atezolizumab		*P**
		Yes	No	
**Total patients, n (%)**	41	10 (24.4%)	31 (75.6%)	
**Age (median, years) (range)**	63.1 (35.1–83.2)	59.5 (43.3–71.3)	63.1 (35.1–83.2)	0.463§
**Sex**				0.123
**Female**	27	9 (90.0%)	18 (58.1%)	
**Male**	14	1 (10.0%)	13 (41.9%)	
**Smoking status**				0.164
**Nonsmokers**	34	10 (100.0%)	24 (77.4%)	
**Smokers**	7	0 (0.0%)	7 (22.6%)	
**ECOG PS**				0.222
**0–1**	31	6 (60.0%)	25 (80.6%)	
**≥2**	10	4 (40.0%)	6 (19.4%)	
**Histology**				1.000
**Nonadenocarcinoma**	2	0 (0.0%)	2 (6.5%)	
**Adenocarcinoma**	39	10 (100.0%)	29 (93.5%)	
**Brain metastasis**				1.000
**No**	16	4 (40.0%)	12 (38.7%)	
**Yes**	25	6 (60.0%)	19 (61.3%)	
**PD-L1 TPS**				0.291#
**<50%**	19	6 (60.0%)	13 (41.9%)	
**≥50%**	6	0 (0.0%)	6 (19.4%)	
**No data**	16	4 (40.0%)	12 (38.7%)	
***EGFR* mutations**				0.548#
**Del-19**	17	5 (50.0%)	12 (38.7%)	
**L858R**	21	5 (50.0%)	16 (51.6%)	
**Other**	3	0 (0.0%)	3 (9.7%)	
**Line of atezolizumab**				0.083
**Second**	9	0 (0.0%)	9 (29.0%)	

**≥Third**	32	10 (100.0%)	22 (71.0%)	
**Atezolizumab therapy**				0.700
**Monotherapy**	11	2 (20.0%)	9 (29.0%)	
**Combination**	30	8 (80.0%)	22 (71.0%)	
**Prior medications**				
**EGFR TKIs**	38	10 (100.0%)	28 (73.7%)	0.564
**ICIs**	2	0 (0.0%)	2 (6.5%)	1.000
**Platinum**	37	10 (100.0%)	27 (87.1%)	0.556
**Pemetrexed**	33	10 (100.0%)	23 (74.2%)	0.165

*Fisher's exact test; [§]Mann–Whitney *U*-test; [#] chi-squared test

ECOG PS, Eastern Cooperative Oncology Group performance status; PD-L1, programmed death-ligand 1; TPS, tumor proportion score.

Only one patient received post- and pre-atezolizumab osimertinib treatment (NTUH-052).

Supplementary Table S4 Multivariate analysis of predictive factors for progression-free survival and prognostic factors for overall survival in patients who received atezolizumab as a second- or subsequent-line treatment.

Prognostic Factors	Multivariate backward LR analysis			
	Progression free survival		Overall survival	
	HR 95% CI	*p*	HR 95% CI	*p*
**Male**				
**Smokers**				
**ECOG PS 2–4**			3.26 (1.55–6.86)	0.002
**Adenocarcinoma**				
**Brain metastasis**				
**PD-L1 TPS**				
≥50% vs. <50%				
No data vs. <50%				
EGFR				
Mutation vs. Wild				
No data vs. Wild				
**≥Third line of atezolizumab**				
**Atezolizumab combination therapy**	0.61 (0.37–1.02)	0.061		
**Prior EGFR TKI**				
**Prior platinum therapy**	0.46 (0.22–0.97)	0.040		
**Prior ICIs**			3.17 (1.19–8.45)	0.021
**Prior pemetrexed**				
**Prior osimertinib**	2.73 (1.05–7.07)	0.039	3.30 (1.16–9.41)	0.026

EGFR, epidermal growth factor receptor gene; EGFR TKI, epidermal growth factor receptor tyrosine kinase inhibitor; ECOG PS, Eastern Cooperative Oncology Group performance status; PD-L1, programmed death-ligand 1; TPS, tumor proportion score; HR, hazard ratio; CI, confidence interval; ICIs, immune checkpoint inhibitors
